# Calcitonin Receptor-Zonula Occludens-1 Interaction Is Critical for Calcitonin-Stimulated Prostate Cancer Metastasis

**DOI:** 10.1371/journal.pone.0150090

**Published:** 2016-03-02

**Authors:** Ahmed Aljameeli, Arvind Thakkar, Shibu Thomas, Vijaybasker Lakshmikanthan, Kenneth A. Iczkowski, Girish V. Shah

**Affiliations:** 1 Pharmacology, University of Louisiana College of Pharmacy, Monroe, LA 71201, United States of America; 2 Pathology, Medical College of Wisconsin, Madison, WI 53226, United States of America; University of Kentucky College of Medicine, UNITED STATES

## Abstract

The role of neuroendocrine peptide calcitonin (CT) and its receptor (CTR) in epithelial cancer progression is an emerging concept with great clinical potential. Expression of CT and CTR is frequently elevated in prostate cancers (PCs) and activation of CT–CTR axis in non-invasive PC cells induces an invasive phenotype. Here we show by yeast-two hybrid screens that CTR associates with the tight junction protein Zonula Occludens-1 (ZO-1) via the interaction between the type 1 PDZ motif at the carboxy-terminus of CTR and the PDZ3 domain of ZO-1. Mutation of either the CTR C-PDZ-binding motif or the ZO-1-PDZ3 domain did not affect binding of CTR with its ligand or G-protein-mediated signaling but abrogated destabilizing actions of CT on tight junctions and formation of distant metastases by orthotopically implanted PC cells in nude mice, indicating that these PDZ domain interactions were pathologically relevant. Further, we observed CTR-ZO-1 interactions in PC specimens by proximity ligation immunohistochemistry, and identified that the number of interactions in metastatic PC specimens was several-fold larger than in non-metastatic PC. Our results for the first time demonstrate a mechanism by which PDZ-mediated interaction between CTR and ZO1 is required for CT-stimulated metastasis of prostate cancer. Since many receptors contain PDZ-binding motifs, this would suggest that PDZ-binding motif-adaptor protein interactions constitute a common mechanism for cancer metastasis.

## Introduction

Calcitonin receptor (CTR) is a member of the class B family of G protein-coupled receptors (GPCRs), which contain numerous drug targets. CTR binds neuroendocrine peptide CT to maintain calcium homeostasis in bone and kidney [1]. However, its expression in multiple organs and its actions in development, cell growth and differentiation suggest that CTR may have more diverse role Tolcos et al. [2],[3,4]. The abundance of CT and CTR transcripts is increased in malignant prostates, and correlates positively with Gleason grade of prostate cancer (PC). In addition, activation of CT-CTR autocrine axis stimulates several processes associated with tumor growth, invasion, angiogenesis, chemoresistance and metastasis, suggesting that CTR serves as an important factor in the progression of a localized PC to its metastatic form [5–7].

CTR mRNA sequence isolated from human prostate lacks a 16-amino acid insert in the first intracellular loop, a characteristic of isoform 2 of CTR [8,9]. CTR2 couples to both stimulatory GTP binding protein G_α_s and G_α_q to co-stimulate adenylyl cyclase and phospholipase C [10]. In addition, CTR destabilizes tight and adherens junctions and activates non-G protein-coupled signaling pathways such as PI-3-kinase (PI3K)-Akt-survivin and WNT/ß-catenin [5,11]. However, the precise mechanism by which CTR stimulates prostate cancer metastasis has not been identified.

Since the disruption of intercellular junctions and acquisition of invasive phenotype are obligate steps in tumor progression, we examined the action of CTR on tight junctions (TJs) and its importance in CTR-stimulated metastasis of PC cells. In this report we show that the cytoplasmic (C) tail of CTR associates with tight junction (TJ) protein Zonula Occludens-1 (ZO-1) via the interaction between the type 1 PDZ-binding motif in the carboxy-terminus of CTR and PDZ3 domain of ZO-1. This interaction is critical for the actions of CTR on TJ destabilization as well as distant metastasis of prostate cancer cells.

## Materials and Methods

### Animals

Male balb/c nu/nu mice (6–8 weeks old) were purchased from Harlan (Madison, WI), and housed two per cage in microisolator units in a barrier facility on a high efficiency particulate arrestance (HEPA)-filtered rack under standard conditions of 12-hour light/dark cycles, fed *ad lib* on a standard autoclaved laboratory diet, and quarantined for one week prior to their use in the study.

### Cell Culture

LNCaP, PC-3 and DU-145 cell lines were obtained from American Tissue Culture Collection (Manassas, VA), and maintained as recommended by the supplier in our laboratory for less than six months after the receipt. PC-31 subline was an isolated PC-3 orthologue that lacked CT and CTR mRNA (S1 Appendix, S1A–S1D Fig). PC-3M cell line was provided by Dr. Isiah Fidler (MD Anderson Cancer Center, Houston, TX). PC-3, PC-31 and PC-3M cell lines were authenticated by STR profiling (S1 Appendix).

### DNA Constructs: FLAGCTRwt and FLAGCTR∆ESS

CTR C-PDZ binding motif (ESSA) was replaced with Alanines by inserting mismatches in respective codons as underlined. The primer sequences were as follows:

Forward Primer: 5’-aag/ctt/atg/gac/tac/aag/gac/gac/gat/gac/aag/agc/ttc/aca/ttt/ aca/agc/cgg/tgc/ttg-3’

Reverse Primers:

CTRwt: 5’-ctc/gag/tca/agc/aga/tga/ctc/ttg/ctc/tat/gat/att/caa/agg/gat/gat/ctc-3’

CTRΔESS: 5’-ctc/gag/tca/agc/agc/agc/agc/ttg/ctc/tat/gat/att/caa/agg/gat/gat/ ctc-3’

The full-length FLAG-CTRwt and FLAG-CTRΔESS were inserted in pcDNA3.1 expression vector by directional cloning (Invitrogen) [12].

### ZO-1∆PDZ mutants

ZO-1 cDNA sequence was mutated by deleting each/or all of the three PDZ domains to obtain the following four mutants: ∆PDZ1 (aa 2–156), ∆PDZ2 (aa 159–252), ∆PDZ3 (aa 294–633) and ∆PDZX (aa 67–1033) [13]. All ZO-1 constructs were myc tagged at the C-terminal and cloned in pCB6 eukaryotic expression vector [13].

The cDNA constructs were transfected in PC-3 cells using FuGene 6™, selected in G418, multiple clones were examined, and those expressing stable transgene expression were selected for subsequent studies as described earlier [11,14].

### Yeast two-hybrid screening

The cDNA sequence corresponding to C-terminal domain of hCTR2 (residues 411–476) was inserted in frame in the pNLex-NLS yeast expression vector [15]. This bait plasmid was used to transform the yeast strain RFY231: *MAT± ura3-1 his3 trp1î*::*hisG*3LexAop-*LEU2*::*leu2*. The transformed yeast was then mated to yeast (RFY309: *MAT*α *his3î200 leu2-3 lys2î201 ura3-52 trp1îhisG*: pSH18-34) pre-transformed with a human prostate cDNA library in pJG4-5 vector (Origene). All rescued colonies were picked and their galactose dependence, leu and lacZ phenotypes were tested [15,16]. ORFs of all galactose-dependent clones were isolated by PCR and cloned into the transcription activation domain (AD) vector by gap repair. The AD clones were then mated against the bait to reconfirm that Leu and lacZ phenotypes were dependent on the ORF. Positive AD clones were further tested for specificity by mating with 7 bait strains expressing unrelated baits (*Campylobacter* proteins) [16].

To check the strength of interaction, original bait strain was mated with strains expressing each isolated AD clone, and reporter activation was scored on 0–3 scale for growth on leu plates (0 = no growth; 3 = maximal growth); and 0–5 scale for lacZ activity on X-Gal plates (0 = white; 5 = dark blue). All interactors in this screen scored maximal growth on leu; and the absence of lacZ activity indicated a weak, but reproducible interaction.

### Cyclic (c) AMP, Protein Kinase A (PKA), and Protein Kinase C (PKC) Assays

PC-3CTR and PC-3CTR∆ESS cells were grown in 24-well plates, washed, and stimulated with various concentrations of CT (0–100 nM) for 3 minutes. The cells were lysed with ice-cold 10% TCA solution, and the lysates were analyzed for cAMP levels by radioimmunoassay.

For PKA and PKC assays, the lysates of untreated/CT-treated cells were incubated with respective PKA or PKC substrate peptide and ^32^P-ATP as described in the protocol provided by the manufacturer (Promega, Madison, WI). In brief, the cells treated with CT or the vehicle for 10 minutes (2 x10^6^ cells per 100-mm dish) were harvested in lysis buffer (50 mM Tris, pH 7.5, containing 5 mM EDTA, 50 mM NaF, 1 mM sodium pyrophosphate, 0.5 mM EGTA, 10 mM ß-mercaptoethanol, 1 μg/ml leupeptin, and 1 μg/ml aprotinin), sonicated, and debris was removed by centrifugation. The extracts were then incubated for 5 min at 30 C in the reaction buffer [final concentration was 50 mM Tris, pH 7.5; 10 mM MgCl_2_; 100 μM ATP; 4 nM of [^32^P]ATP; 0.25 mg/ml BSA; and 50 μM PKA or PKC substrate peptides (Promega Corp., Madison, WI)]. 10 μM cAMP (total PKA activity), served as a positive control and 1 μM PKI plus cAMP served as a negative control for PKA assays (total background activity). Triplicates of each sample were assayed, and blotted on SAM2 biotin capture membranes at the end of incubation (Promega Corp., Milwaukee, WI). The membranes were then washed extensively with 2 M NaCl as well as 2 M NaCl 1% H_3_PO_4_, and the bound phosphorylated substrate on a filter disc was quantified in a scintillation counter. PKI-inhibitable kinase activity was calculated, and the data were reported as picomoles ATP per min per g protein.

### Immunofluorescence

The cells (approximately 5x10^4^ cells) were cultured either on transwell chambers (0.4μm pore size) and grown to confluency (approximately 5 days). The cells were then fixed, and immunolabeled with the appropriate primary antibody (mouse Anti-CTR, Acris Laboratories; rabbit Anti-ZO-1 serum, Invitrogen; rabbit anti-claudin 3 antiserum, Invitrogen) for overnight at 4^0^ C. The corresponding secondary antibodies conjugated with fluorophores were applied. After the washes, the slides were observed under Nikon Optiphot-2 microscope equipped for epifluorescence. The images were captured with Retiga 1300 camera connected to an iMac computer loaded with iVision image analysis program (Biovision Technologies, Exton, PA). All antibodies were characterized for specificity and cross-reactivity as described in S1 Appendix, S2A–S2C Fig.

### Measurement of Transepithelial Electric Resistance (TER) and Paracellular Permeability (PCP)

Approximately 5x10^4^ cells were plated on Transwell filters (0.4μm pore size) and grown to confluency (usually 5–6 days). TER (in duplicate wells) was measured at several time intervals with EVOM volt-ohm meter as previously described [11]. The readings were corrected for the blank (TER values of the filter in bathing medium). The integrity and cell density of monolayers were carefully monitored.

For PCP measurements, cells were grown to confluence on Transwell filters. Tetra methyl rhodamine-dextran (1 mg/ml, ∼ 4 kDa, Sigma) was added to the upper chamber. Fluorescence of the lower chamber medium was measured in Modulus Microplate Reader (Turner Biosystems) after one hour.

### *In vitro* Invasion Assay

Invasion experiments were conducted in 24-well, two compartmented, Matrigel™ invasion chambers as previously described [17].

Growth Correction: Since CT also induces proliferation of PC cell lines, we determined the factor to correct for possible proliferation of cells that may have migrated in early phase of 24 h incubation period. 25 x 10^4^ cells were plated at hourly intervals in six-well dishes and cultured for 1–24 hours. Mean percent increase in cell number in each well was determined. This correction was applied to the results of invasion assays.

### CTR-C Pull-down Assay

cDNA encoding the intracellular domain of CTRwt and CTR∆ESS (aa 441–476) was cloned into pGEX vector (GE Healthcare, Piscataway, NJ). Control (C)-cDNA construct (random cDNA sequence of equivalent length) was prepared for a negative control. The cDNAs were expressed in BL-21 *Escherichia coli* strain, and equivalent amount of each fusion protein (approx 2 mg) was absorbed on glutathione-Sepharose 4B (GE Healthcare). The beads containing GST-Fusion proteins of C peptide, CTR-Cwt and CTR-C∆ESS were then incubated with PC-31 cell lysate. The bound proteins were eluted with reduced glutathione. The presence of ZO-1 in the eluate was detected by Western blotting.

### Overlay assays

Overlay assays were performed as previously described [18]. Cell lysates from stable PC-3M transfectants expressing either ZO-1wt-MYC or ZO-1∆PDZ-MYC mutants were prepared, fractionated on an SDS-PAGE gel and transferred to nitrocellulose membranes. The blot was cut into strips representing each lane of the gel. The strips were then blocked in far Western buffer and incubated with purified GST tagged CTRwt fusion protein overnight at 4°C, and processed for GST immunoblotting. For protein loading controls, the blots were stripped and reprobed for MYC.

### Co-immunoprecpitation

Serum-starved PC-31-V, PC-31-CTRwt or PC-31-CTR∆ESS cells (2 x 10^6^ per 100 mm dish) were stimulated with/without 50 nM CT for 3 minutes, washed, and lysed in the IP buffer as previously described [19]. CTR in normalized lysate proteins was immunoprecipitated with anti-FLAG EZ-view beads (Sigma), and eluted with FLAG peptide (600 μg/ml in PBS). CTR immunopreciptates were processed for ZO-1 immunoblotting. To control for protein loading and transfer, the blots were stripped and reprobed with immunoprecipitating antibody.

### Acceptor Photo-bleaching Fluorescence Resonance Energy Transfer (FRET) Microscopy

PC-31 cells were plated on glass bottom chambers and grown overnight. The cells were transfected with CTRwt-ECFP-N1 (or CTR∆ESS-ECFP-N1) and ZO-1wt-EYFP-N1 (or ZO1-∆PDZ3-EYFP-N1) plasmids using Lipofectamine™, cultured for additional 36 h, and imaged live after treatment with 50 nM CT (or control) at room temperature using Nikon Eclipse 2000 TE connected with Sensicam-qe (Cooke Corporation, Romulus, MI). CFP and YFP images were acquired with excitation at 436 nm and emission at 480/535 nm respectively. A 505 nm dichroic filter set in Dual View was used to split the images (Optical Insights, Santa Fe, NM). FRET was recorded by monitoring the quenching of CFP during YFP photobleaching. The images were analyzed with IPLab 4.0 imaging software (Scanalytics, Inc, Rockville, MD) and FRET efficiencies were calculated using the formula: FRET_Efficiency_ = (donor CFP_after bleaching_—donor CFP_before bleaching_)/donor CFP_after bleaching_. Thresholds for donor (CFP), acceptor (YFP), and FRET images were set at the beginning of data collection and were kept unchanged for the entire data set.

### *In situ* proximity ligation assay (PLA)

Fixed PC cells or tissues were immunolabeled with primary antibodies (anti-rabbit ZO-1 serum and anti-goat CTR serum) for overnight at 4°C. The secondary antibodies with attached PLA probes are supplied in the Duolink™ kit (Olink Bioscience, Uppsala, Sweden). CTR-ZO-1 interaction was observed by confocal microscopy at 400X. A red fluorescent dot indicates the two proteins are within 35nm distance. The number of dots per cell was determined by Blobfinder™ image analysis software [20].

### Frozen Prostate tumors

Snap-frozen human prostate tumor specimens were obtained from Louisiana Cancer Research Center, and were processed for co-immunoprecipitation as described previously [21]. The human prostate cancer tumor tissues were obtained from the Louisiana Cancer Research Consortium (LCRC; New Orleans, LA) with written patient consent and the Institutional Review Board approval of both institutions, IRB of the University of Louisiana at Monroe and Tulane University Research Committee (TURC) of Tulane University Medical Center and Louisiana Cancer Research Center. For a similar study see Liu et al [22].

### Orthotopic tumor growth and metastasis

To detect micrometastases of implanted tumor cells in mice, we stably transfected the cell lines with DsRed-MCherry-Hyg-N1 (Clontech, Palo Alto, CA). Hygromycin resistant colonies that expressed strong red fluorescence at a steady level over the observation period were selected, and used for orthotopic implantation.

All animal procedures were conducted in accordance with the principles and procedures outlined by the NIH and Institutional Animal Care and Use Committee at University of Louisiana at Monroe. The animal procedures used in this study were approved by the IACUC of University of Louisiana at Monroe. The orthotopic implantation surgery was performed under Ketamin/Xylazine anesthesia as previously described [6]. Tumor cell suspensions (1x10^6^ cells/20μl) were injected into the dorsal prostate, the animals were monitored for tumor growth and metastasis by fluorography using Kodak 4000 MM imaging station, and sacrificed within sixty days [23]. The animals were observed on a daily basis and humanely sacrificed by CO_2_ inhalation when they met the following humane endpoint criteria: prostration, skin lesions, significant body weight loss, difficulty breathing, epistaxis, rotational motion and body temperature drop. Several organs were collected, fixed and examined for tumor metastasis. Euthanasia was performed by trained investigators Drs. Arvind Thakkar and Vijaybasker Lakshmikanthan. Dr. Shibu Thomas was involved in early part of the study where he contributed to the development of constructs, generation of cell lines and establishment of orthotopic implantation model.

At necropsy, primary tumor and other organs were harvested and weighed. Wet sections of organs were examined for the presence of RFP. The remaining tissues were fixed in neutral buffered formalin and processed for H&E staining. The use of animals was necessary to evaluate tumorigenicity and metastastasizing ability of cell lines expressing wild type or mutant CTR.

Male balb/c nu/nu mice (6–8 weeks old) were purchased from Harlan (Madison, WI), and housed two per cage in microisolator units in a barrier facility on a high efficiency particulate arrestance (HEPA)-filtered rack under standard conditions of 12-hour light/dark cycles, fed *ad lib* on a standard autoclaved laboratory diet, and quarantined for one week prior to their use in the study.

## Results and Discussion

### CTR cytoplasmic (C) tail contains a type I PDZ-binding motif

Several class B GPCRs activate non-G protein-mediated signaling by interacting with scaffolding proteins through their cytoplasmic tails [24–26]. Since CTR also activated non-G protein-coupled signaling, we examined its primary sequence and found that the four terminal residues of CTR C-tail (aa 471–74, E-S-S-A) formed a canonical type I PDZ domain-binding motif [27,28]. We inactivated this motif by replacing these residues with Alanine (referred as ∆ESS).

**Fig 1:**
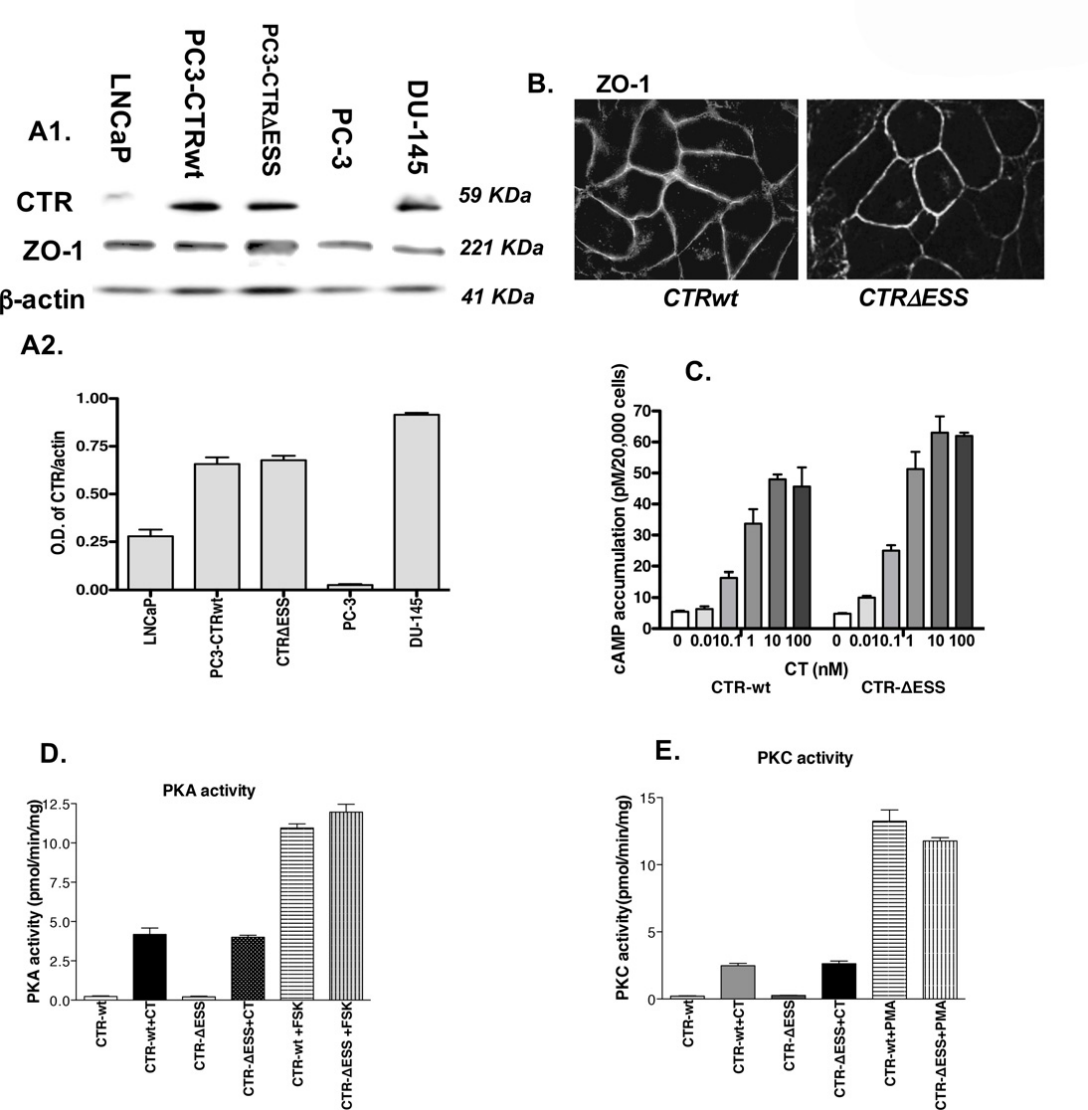
Mutation of CTR-C tail does not affect its localization or signaling ability. **A1.** CTR immunoreactivity in plasma membranes of PC cell lines (30 μg protein/lane) was determined by Western blot analysis. The blot was reprobed for ß-actin for normalization. Representative of three separate experiments. **A2**. Optical Density of CTR and actin bands on each blot was obtained by image analysis on Kodak 4000 MM Image station. The figure presents the group data (ratio of CTR OD/Actin OD ± SEM of 3 blots). **B.** PC-3-CTRwt and PC-3CTR-∆ESS cells were cultured in transwell chambers to confluence, washed, fixed and processed for CTR immunofluorescence as described in Methods. The photomicrographs are 400X. **C**. PC-3CTR and PC-3CTR∆ESS cells were stimulated with various concentrations of CT (0–100 nM) for 3 minutes. The cells were lysed, and the lysates were analyzed for cAMP levels by radioimmunoassay. **D-E.** Lysates of untreated/CT-treated PC-3CTRwt and PC-3CTR∆ESS cells were incubated with respective PKA or PKC substrate peptide and ^32^P-ATP as described in the protocol provided by the manufacturer (Promega, Madison, WI). Amount of ^32^P-labeled substrate peptide was determined, and the activity was calculated.

PC-3 cells were chosen because they lack endogenous CTR and enable the study of the expressed CTR without interference from endogenous CTR. We compared the levels of CTRwt or CTR∆ESS in plasma membranes of PC-3 stable lines with endogenous CTR levels in plasma membranes of other PC cell lines. PC-3 cells lacked CTR, and LNCaP cells displayed low levels of CTR. However, the levels of CTR in PC-3CTRwt and PC3-CTR∆ESS cells were comparable to endogenous CTR levels of poorly-differentiated DU-145 cell line as detected by immunoblotting (Fig 1A1 and 1A2).

### Mutation of CTR-C PDZ-binding motif does not alter subcellular localization of CTR or its G protein-coupled signaling

Both CTRwt and CTR∆ESS were localized to the plasma membrane (Fig 1B). To examine the ability of CTRwt and CTR∆ESS to activate its G protein-coupled effectors, we examined the whole dose-response curve of CT on cAMP accumulation in CTRwt and CTR∆ESS cell lines, and analyzed the effect of 50 nM CT on PKA and PKC activity. Both receptors caused a similar increase in cAMP accumulation over a concentration range of 0–100 nM and induced similar increases in PKA and PKC activity (Fig 1C–1E). Taken together, these results suggest that ∆ESS mutation does not alter either the localization of CTR on PC-3 cell membranes or its ability to activate G_α_s and G_α_q signaling.

### CTR-C PDZ-binding motif is required for the action of CTR on TJ stability and invasion

#### A. TER

**Fig 2:**
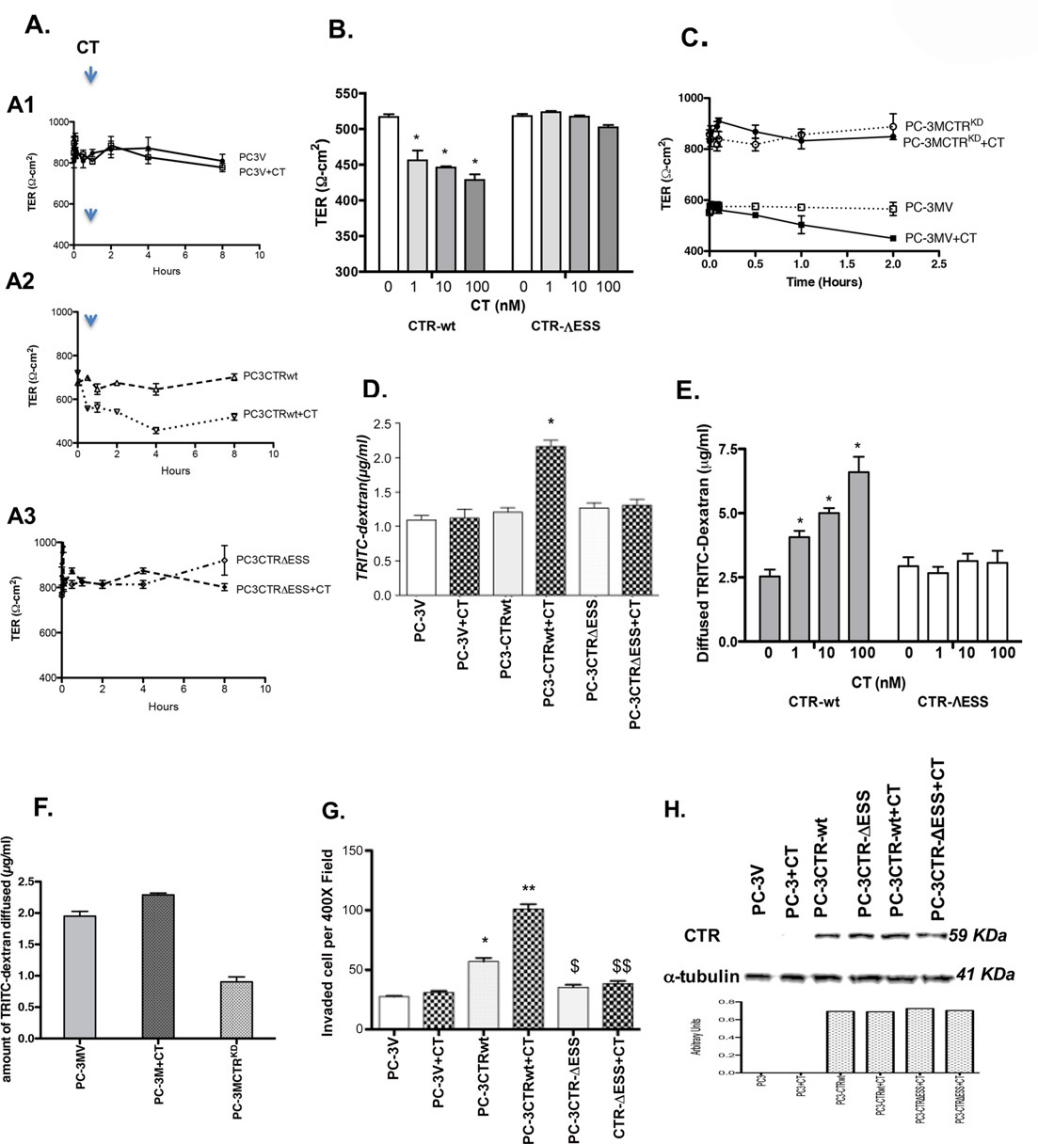
CTR-C PDZ- binding motif mutation and TJ disruption and invasion. A. Transepithelial Electric Resistance (TER): Polarized PC-3v, PC-3-CTRwt and PC-3CTR-∆ESS cells were serum-starved for 4 hours, then treated with/without 50 nM CT. TER was measured with EVOM volt-ohm meter. The results are expressed as ohm-cm2± s.e.m. for n = 6). B. Polarized PC-3-CTRwt and PC-3CTR-∆ESS cells were serum-starved for 4 hours, then treated with/without various concentrations of CT 0–100 nM). TER was measured two hours after the addition of CT with EVOM volt-ohm meter. The results are expressed as TER (ohm-cm2) ± s.e.m. for n = 6. C. PC-3M cells were stably transfected with either carrier plasmid pSuper.neo expressing scrambled RNA (PC-3MV) or pSuper.neo vector expressing CTR shRNA (PC-3MCTR^KD^) [14,42]. The cells were cultured as described in Fig 2A and TER was measured at various time intervals upto 2 hours in the presence of absence of 50 nM CT. The results are expressed as mean ± s.e.m. for n = 4. Knock-down of CTR alone caused a significant increase in TER of PC-3M cells, demonstrating the importance of endogenous CTR in regulating TJ stability of PC-3M cells. D. Paracellular Permeability (PCP): Polarized PC-3V, PC-3-CTRwt and PC-3CTR∆ESS cells were treated with/without 50 nM CT. PCP was determined by diffusion of ~4kDa TRITC-conjugated dextran from the upper to the lower chamber in one hour. The results are expressed as μg/ml/hour of TRITC-Dextran diffused ± s.e.m. for n = 6. *p<0.05 (-CT vs +CT, one -way ANOVA and Newman-Keuls test). E. In an experiment similar to 2B, PCP was determined by diffusion of ∼ 4kDa TRITC-conjugated dextran from the upper to the lower chamber in two hours. The results are expressed as μg/ml/hour of TRITC-Dextran diffused ± s.e.m for n = 6. *p<0.05 (-CT vs +CT, One-way ANOVA and Newman-Keuls test). F. PC-3MV and PC-3MCTR^KD^ cells were tested for PCP as described in Fig 2C. Again, the knock-down of CTR produced a significant decrease in PCP of PC-3M cells, demonstrating the importance of endogenous CTR in regulating TJ stability of PC-3M cells. The results are expressed as μg/ml/hour of TRITC-Dextran diffused ± s.e.m for n = 6. *p<0.05 (-CT vs +CT, One-way ANOVA and Newman-Keuls test). G. Invasion: PC-3V, PC-3CTRwt and PC-3CTR∆ESS cells were added to upper insert of invasion chambers along with/without 50 nM CT, and cells that passed through the Matrigel™ barrier™ were counted after 48 h. The results are expressed as mean number of invaded cells per 400X field ± s.e.m. for n = 6. *p <0.05 (PC-3V vs. PC-3CTR-wt); $ p <0.05 (PC-3CTR-wt vs. PC-3CTR∆ESS); $ $-p <0.05 (PC-3CTRwt+CT vs. PC-3CTR∆ESS+CT) (One-way ANOVA and Newman-Keuls test). H. CTR immunoreactivity in PC-3V, PC-3-CTRwt and PC-3CTR∆ESS cells. The cells were treated with/without 50nM CT. CTR in normalized lysate proteins was quantitated by immunoblotting. Representative blot of three separate experiments.

To examine the effect of CT on tight junctions (TJs), TER of polarized PC-3 cells expressing either the vector (PC-3V), CTRwt or CTR∆ESS was monitored for 8 hours after CT stimulation. Polarized PC-3V cells displayed relatively high TER, and CT did not alter it (Fig 2A1). In contrast, the expression of CTRwt resulted in a 40% reduction of TER in PC-3CTRwt cells (Fig 2A2). This drop is most likely triggered by interaction of the transfected CTR with low levels of CT normally secreted by PC-3 cells. Indeed, the addition of exogenous CT (50 nM) decreased TER even further. However, the expression of CTR∆ESS in PC-3 cells did not affect TER either before or after the addition of exogenous CT (Fig 2A3). We chose 50 nM dose of CT based on our earlier studies showing 10- to 20-fold increase in endogenous CT levels as well as CT-secreting cell populations of malignant prostates [29,30]. Moreover, CT was maximally effective at 50 nM dose in stimulating invasion of PC cells [17]. We also tested CT at lower concentrations. We observed that CT significantly decreased TER of PC-3CTRwt cells at the concentrations of 1 nM or greater when incubated for 2 hours (Fig 2B). Again, PC-3CTR∆ESS cells did not show any changes in TER at all CT concentrations under those conditions (Fig 2B). To test the role of endogenous CTR in TJ stability, we used PC-3M cell line, which is derived from PC-3 cells, is highly aggressive, and co-expresses CT as well as CTR [30,31]. The knock-down of endogenous CTR alone induced a significant increase in TER of PC-3M cells (Fig 2C).

#### B. PCP

TJs form a barrier that regulates the movement of ions, solutes and growth factors between cells. To detect whether CT affects the ‘leak pathway”, i.e. the barrier to large uncharged molecules, we measured diffusion of fluorophore-conjugated dextran across polarized PC cell line monolayers. PC-3V cells displayed relatively low PCP and exogenous CT did not alter it (Fig 2D). Expression of CTRwt in PC-3 cells caused a small increase in their PCP, and the addition of CT increased PCP significantly. In contrast, CTR∆ESS expression had little effect on PCP in the presence or absence of CT. When tested at multiple CT concentrations, CT induced an increase in PCP of PC-3CTRwt cells at the doses of 1 nM or higher after 2 hours incubation (Fig 2E). Again, CT did not alter PCP of PC-3CTR∆ESS cells at all tested concentrations (Fig 2E). To examine the importance of endogenous CTR in TJ stability, we again used PC-3M cells [30]. As expected, CTR^KD^ significantly decreased PCP of PC-3M cells (Fig 2F). When considered together, these results suggest that CT-CTR axis may be involved in dynamic regulation of TJs in prostate cells.

#### C. Invasion

CT increases invasiveness, and even induces an invasive phenotype in non-invasive PC cells [14]. We examined the role of PDZ-binding motif in CT-stimulated invasion. Expression of CTRwt in PC-3 cells increased invasion by 2.5-fold (Fig 2G). However, the expression of CTR∆ESS did not cause a similar increase. When stimulated with 50 nM CT, PC-3-CTRwt cells responded with an additional 2.5-fold increase in invasion, but PC-3CTR∆ESS did not. Since CTRwt and CTR∆ESS had dramatically different effects on TER, PCP and invasion, we wanted to make certain that the variances were not due to the differences in their CTR levels. Immunoreactive CTR levels were similar in all cell lines in the presence or absence of CT (Fig 2H), which suggests that the interaction of CTR with its partner protein through its PDZ binding motif is critical for its destabilizing actions on TJs and promoting invasion

### CTR also destabilizes TJs and increases invasion of other PC cell lines

**Fig 3:**
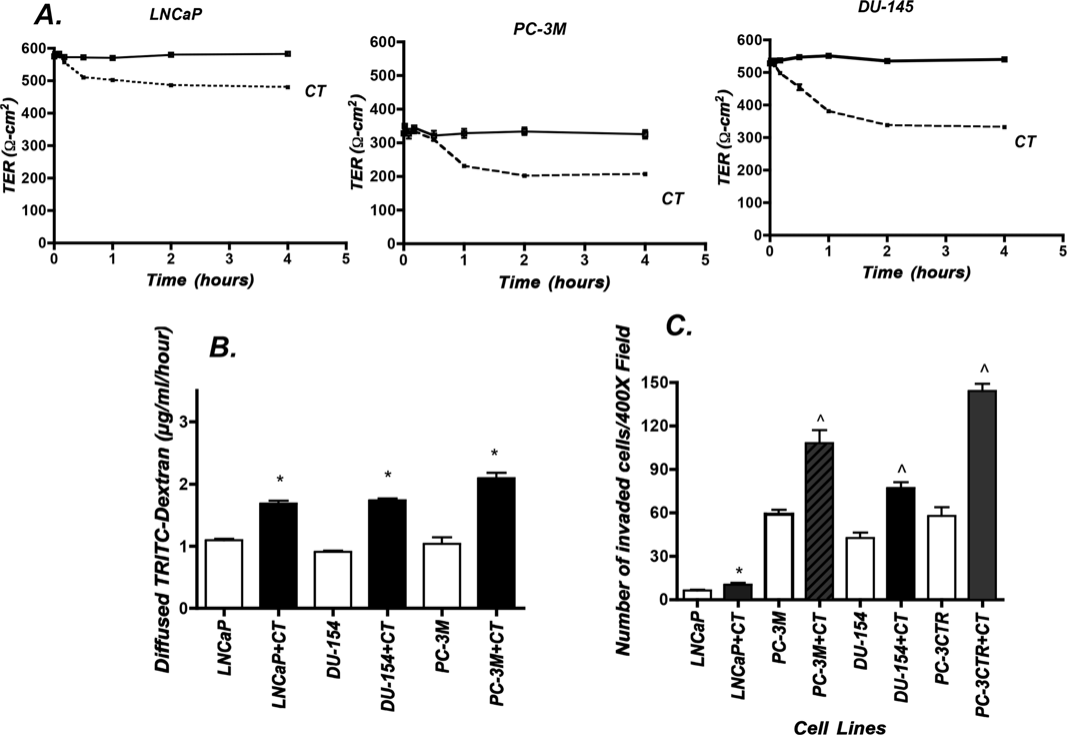
Effect of CT on TER, PCP and Invasion of other PC cell lines. LNCaP, PC-3M and DU-145 cells were treated with 50 nM CT, and their TER (A), PCP (B) and invasion (C) were measured as described in Methods. Results are presented as mean ±SEM (n = 6). *p < 0.05 from corresponding control (-CT vs +CT, One-way ANOVA and Newman Keuls test). ^p<0.01 (-CT vs +CT, One-way ANOVA and Newman-Keuls test).

We also tested whether CT produces similar effects in LNCaP, PC-3M and DU-145 cell lines, which express endogenous CTR (Fig 1A). CT reduced TER and increased PCP of these cell lines, suggesting CT destabilizes TJs in androgen-responsive as well as androgen-refractory PC cell lines (Fig 3A and 3B). Similarly, CT significantly increased invasion of less invasive LNCaP as well as more invasive DU-145 and PC-3M cells (Fig 3C).

### Identification of CTR-interacting protein

Since the action of CTR on TJ stability and invasion required PDZ-binding motif, we sought to identify CTR-interacting protein by Y2H complementation screening. After screening 1x10^6^ transformants, 11 interacting clones were identified, purified and sequenced (Table 1). Most positive clones encoded plasma membrane proteins and/or proteins associated with the adenylyl cyclase system. As a novel finding, one clone encoded tight junction protein ZO-1, and this clone also provided the strongest signal in secondary screening. Since CTR destabilized TJs only in the presence of PDZ-binding motif, we characterized CTR-ZO-1 interaction further.

**Table 1:** 

No	Gene	X-Gal Score	Leu Score
1	PP1R9A-ERM Domain	++	+++
2	PDE9Av20	+	+++
3	Tight junction protein 1(TJP-1) (Zonula Occludens-1)	+++	+++
4	SNX4-nexin-4	++	+++
5	Apoptosis-inducing Factor-PCDC8	++	+++
6	Vigilin-high density lipoprotein binding protein	+	+++
7	SMARCA4	++	+++
8	MAPK9v2	++	+++
9	Homo sapiens tumor-associated calcium signal transducer 2 (TACSTD2)	++	+++
10	Homo sapiens transgelin (TAGLN) transcript variant 2 mRNA	+	+++
11	Homo sapiens WNK lysine deficient protein kinase 4 (WNK4), mRNA	++	+++

### CTR-C-tail interacts with ZO-1: A. Pull-down assay

**Fig 4:**
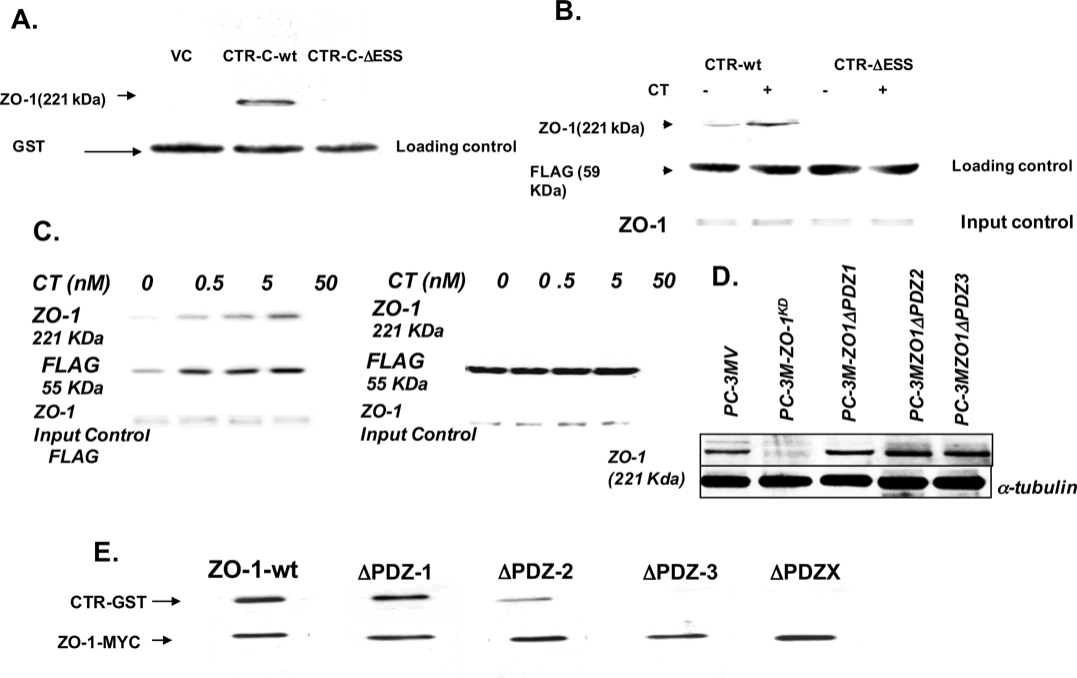
Validation of CTR-ZO-1 interaction. **A. FLAG-CTR-C-pull down.** The beads containing GST-fusion proteins of C, CTR-Cwt and CTR-C∆ESS (2 μg proteins) were incubated with PC-3 cell lysates for 6 h at 4^o^ C, washed, and bound proteins were eluted with 5 mM reduced glutathione. ZO-1 in eluted proteins was detected by immunoblotting. Representative blot of three separate experiments. **B**. **Co-precipitation of CTR-FLAG with ZO-1.** PC-31FLAG-CTRwt and PC-31FLAG-CTR∆ESS cells were treated with 50 nM CT for 3 minutes. CTR-FLAG was immunoprecipitated, the IPs were fractionated on SDS-PAGE and ZO-1 was detected by immunoblotting. Representative blot of three separate experiments. **C.** We used 50 nM CT as a standard CT concentration because that dose of CT produced a maximal increase in invasion of PC cell. However, we also examined the ability of CTR to co-IP ZO-1 at lower CT concentrations. We tested the effects of 0.5 nM, 5 nM and 50 nM on CTR-ZO-1 association. CTRwt could co-IP ZO-1 in small amounts even in the absence of exogenous CT. Addition of 0.5 nM or higher CT observably increased CTR-ZO-1 association as assessed by co-IP of ZO-1in a dose-dependent fashion. In contrast, CTR∆ESS failed to co-IP ZO-1 at all CT concentrations. **D.** PC-3M cells stably transfected with pSuperNeo expressing a scrambled control (SC) or ZO-1 siRNA, were again transfected with ZO-1wt or its ∆PDZ mutants. Cell lysates were tested for ZO-1 or mutants by ZO-1 immunoblotting. **E. Overlay Assays**: Lysates of cells expressing either ZO-1wt-MYC or ZO-1∆PDZ-MYC (∆PDZ-1 denotes PDZ-1 mutated with other PDZ domains intact) were loaded on a gel. Blots made by transferring the proteins from gel were incubated with GST tagged CTR-wt fusion protein overnight at 4°C. The blots were then washed and immunoprobed for GST. The same blots were then stripped and reprobed for MYC protein. Bands appeared at the same place both times. Representative blots of three separate experiments.

CTR-C tail-ZO-1 interaction was examined using GST pull-down assays. Equal amounts of GST-C (negative control), GST-CTR-Cwt and GST-CTR-C∆ESS fusion proteins (2 μg) were immobilized on gluthatione-Sepharose 4B beads, and incubated individually with equal amount of PC-3 cell lysates. Bound proteins were analyzed for ZO-1 immunoreactivity by Western blotting. Only CTR-C-wt fusion protein, but not CTR-C∆ESS or–C, pulled ZO-1 immunoreactivity down (Fig 4A).

### B. Co-precipitation

To confirm CTR and ZO-1 interaction in a more physiological context, co-immunoprecipitation studies were performed. To avoid activation of CTR by endogenous CT in PC-3 cells, we used the PC-31 cell line that lacks endogenous CT and CTR. PC-31 cells stably expressing either FLAG-CTRwt or FLAGCTR**∆**ESS were treated with or without CT for 3 minutes. The analysis of FLAG-CTR IPs confirmed that only FLAG-CTRwt, but not FLAG-CTR∆ESS, co-precipitated ZO-1 (Fig 4B). Interestingly, ligand-activated FLAG-CTRwt precipitated ZO-1 much more efficiently than dormant FLAG-CTRwt. The mechanism could involve a ligand-induced conformational or post-translational modification, but was not further characterized in the current study. We also tested the effect of multiple CT concentrations on CTR-ZO-1 co-precipitation in PC-3 cells. As presented in Fig 4C, it appears that co-precipitation of ZO-1 by CTRwt was less in the presence of 1nM CT, but increased with increasing CT concentrations. However, CTR∆ESS could not co-immunoprecipitate ZO-1 at all CT concentrations (Fig 4D). The results confirm the role of the ligand in the activation of CTR and in enhancing CTR-ZO-1 interaction; and explain the results of Fig 2.

### Identification of CTR-C interaction site on ZO-1: blot overlay assay

Since ZO-1 contains three PDZ domains, we wished to identify which of these interacts with CTR-C PDZ-binding motif. We used PC-3M cells for this study because they are PC-3-derived, but co-express CT and CTR, and are suitable for monitoring the modulation of CTR action in response to mutations in ZO-1 [30]. To knock down the endogenous ZO-1 expression, PC-3M cells were stably transfected with constitutive ZO-1 shRNA expression vector (pSuper.neo system; shRNA duplex- 5’-GACGAGAUAAUCCUCAUUUtt-3’ corresponding to 596–614 bp of ZO-1 mRNA). To overcome the constitutive presence of ZO-1 shRNA in PC-3M cells that can interfere with the reexpression of ZO-1 wt or its ∆PDZ mutants, we generated ZO-1 (wt or ∆PDZ mutants) cDNA expression vectors refractory to ZO-1 RNAi by introducing a silent mutation in RNAi target sequence in the ZO-1 transgene. The cells were then transfected with either ZO-1-wt or a ZO-1-∆PDZ transgenes, where either one (∆PDZ1, ∆PDZ2 or ∆PDZ3) or all three PDZ domains [∆PDZX] were deleted. Knock-down of ZO-1 as well as its reexpression in PC-3M cells was verified by Western blotting (Fig 4D). The lysates from these cells were fractionated on SDS-PAGE and transferred to PVDF membrane. After incubation with GST-CTRwt fusion protein, the blots were probed first for GST and then for MYC immunoreactivity (Fig 4E). ZO-1-wt as well as ZO-1-∆PDZ1 and ∆PDZ2 bound CTRwt-GST. However, deletion of either PDZ3 alone (∆PDZ3) or all three PDZ domains (∆PDZX) abolished the ability of ZO-1 to bind to CTRwt-GST, suggesting CTR-C interacts with ZO-1 at its PDZ3 domain.

### CTR-C PDZ-binding motif-ZO-1-PDZ3 interaction in live PC cells: acceptor photobleaching FRET Microscopy

**Fig 5:**
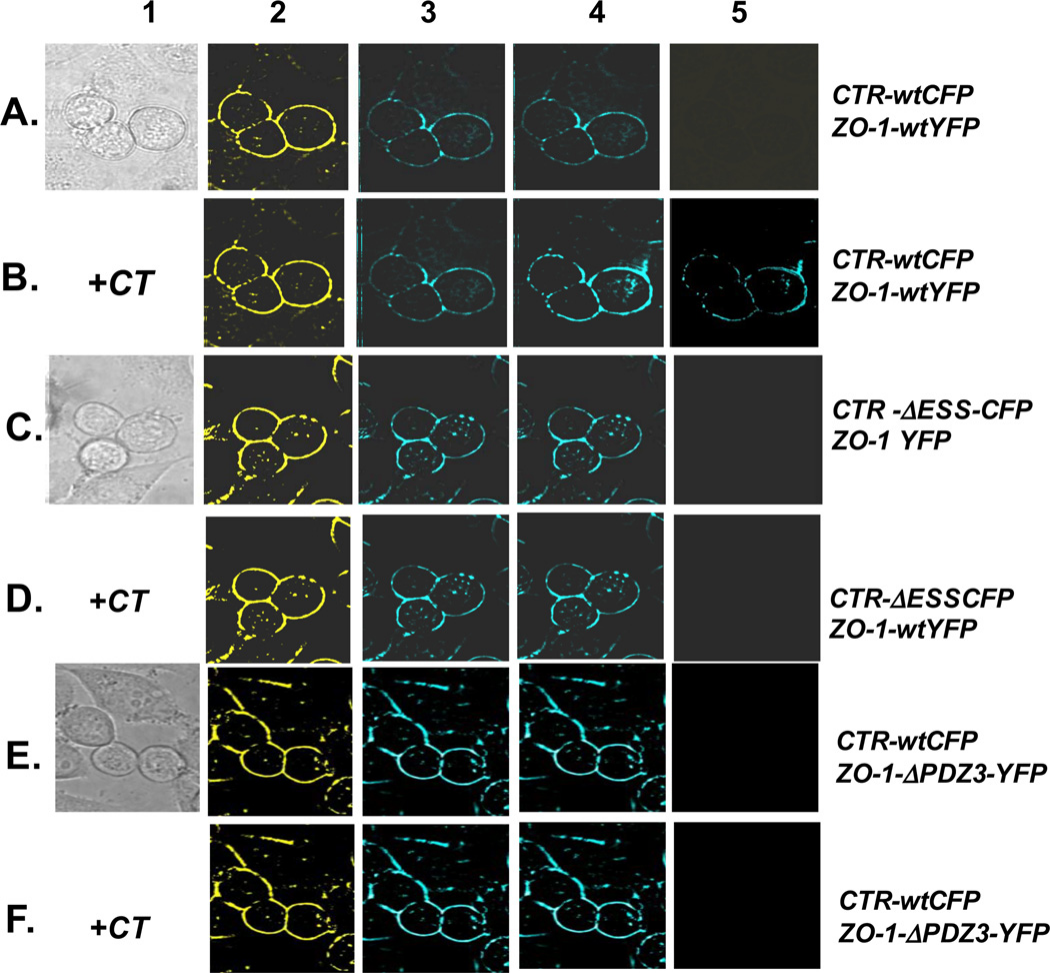
Acceptor photobleaching FRET microscopy. Live PC-31 cells co-expressing CTR-wtCFP and ZO-1-wtYFP were imaged at room temperature with excitation at 436 nm. CFP and YFP emissions were monitored at 480 nm and 535 nm respectively. Micrographs under column 1 are differential interference contrast image of cells in culture. Micrographs under columns 2 and 3 are YFP and CFP images pre-bleaching. Micrographs under column 4 are images of CFP post-YFP bleach, and micrographs under column 5 = column 4—column 3. **A and B:** Experiments with FRET pair CTRwt-CFP:ZO-1wt-YFP in absence/presence of CT. **C and D:** Experiments with FRET pair CTR∆ESS-CFP:ZO-1wt-YFP in absence/presence of CT. **E and F**: Experiments with FRET pair CTRwt-CFP:ZO-1∆PDZ3-YFP in absence/presence of CT.

To verify CTR and ZO-1 interaction in live cells, we employed acceptor photobleaching FRET microscopy [32]. Both ZO-1 and CTR localized to the plasma membrane (Fig 5A, Columns 2 and 3 respectively). An increase in the intensity of CFP with YFP bleaching suggested energy transfer or interaction; no change in CFP intensity suggested a lack of interaction. The results of Fig 5B show that the addition of CT (50 nM) caused a visible increase in CFP intensity (B5 = B4—B3), suggesting an interaction of CTR with ZO-1. Mean FRET efficiency of the resting CTR-wt-ZO-1-wt pair was 8.419±0.962 (n = 8 separate experiments) suggesting a weak interaction (Fig 5A). CT stimulation increased mean FRET efficiency to 97.38 ± 10.04, suggestive of moderately strong interaction (Fig 5B). However, neither the CTR-∆ESSCFP:ZO-wt-YFP nor CTR-wt-CFP:ZO-1-∆PDZ3-YFP FRET pairs demonstrated any interaction in the presence or absence of CT (Fig 5C–5F). These results again confirm that the interaction of CTR with ZO-1 requires the CTR-C PDZ-binding motif and the ZO-1-PDZ3 domain. Activated CTRwt displayed stronger interaction with ZO-1 than the resting CTRwt, consistent with the data of co-immunoprecipitation presented in Fig 4B.

### ZO-1-PDZ3 deletion abrogates the action of CTR on TJs and invasion

**Fig 6:**
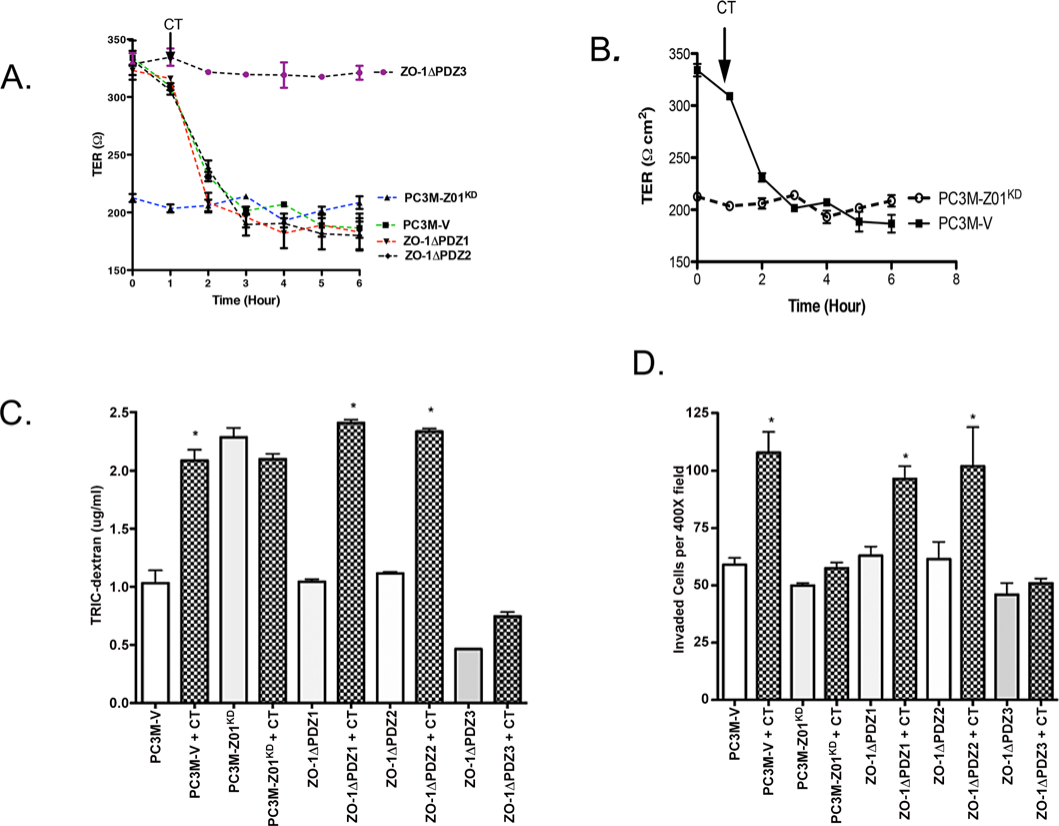
ZO-1 mutants and CTR-stimulated TER, PCP and invasion. **A**. **Transepithelial Electric Resistance:** TER of polarized PC-3M cells or those expressing ZO-1 mutants (as labeled-either ZO-1_KD_, ∆PDZ1, ∆PDZ2 and ∆PDZ3) was measured at several time points after stimulation with 50 nM CT (ohm-cm^2^± s.e.m. for n = 6). PC-3M cells used in these studies. **B.** Comparison of TER displayed by PC-3M cells vs PC-3M-ZO-1_KD_ cells under the experimental conditions of Fig 5B. **C**. **Paracellular Permeability**: PCP of polarized PC-3M cells or those expressing ZO-1 mutants (as labeled) was measured as described in Fig 1B. The results are expressed as μg/ml/hour of TRITC-Dextran diffused ± s.e.m. for n = 6. *p<0.05 significantly different from PC-3M V cells (One-way ANOVA and Newman-Keuls test). **D**. **Invasion**: PC-3M cells or those expressing ZO-1 mutants (as labeled) were treated with/without 50 nM CT, and cells that passed through the Matrigel barrier™ were counted after 48 hours. The results are expressed as mean number of invaded cells per 400X field ± s.e.m. for n = 6. *p<0.05 significantly different from PC-3MV cells (One-way ANOVA and Newman-keuls test).

First, we verified the expression of ZO-1 protein in PC-3M sublines that expressed either ZO-1-wt or its ∆PDZ mutants (Fig 4D). As expected, ZO-1_KD_ cells lacked ZO-1 expression, whereas other sublines displayed ZO-1 expression; but because of lower molecular weights, ZO-1-∆PDZ variants displayed slightly more electrophoretic mobility as compared to ZO-1wt. To determine the functional significance of each ZO-1 PDZ domain in CTR-ZO-1 interaction, we examined the effect of CT on TJs and invasion in cells expressing ZO-1-wt or its ∆PDZ mutants. PC-3M-ZO-1^KD^ cells had a lower TER relative to PC-3M-V cells (vector control) or those reexpressing ZO-1-wt or its ∆PDZ variants (Fig 6A and 6B). Reexpression of ZO-1wt or its ∆PDZ variants restored their TER closer to that of PC-3M-V cells. Moreover, the cells reexpressing ZO-1-wt or its ∆PDZ1 and ∆PDZ2 variants responded to CT with a remarkable but similar decline in TER. In contrast, the cells expressing ZO-1-∆PDZ3 showed no decrease in TER in response to CT (Fig 6B). A similar trend was observed with PCP and invasion (Fig 6C and 6D respectively). These results corroborate the findings of Figs 3C and 4F that the loss of PDZ3 domain of ZO-1, but not PDZ1 or 2 domains, abrogates CT-stimulated TJ destabilization and invasion.

### CTR-ZO-1 interaction is observed in PC cell lines and tumor specimens

**Fig 7:**
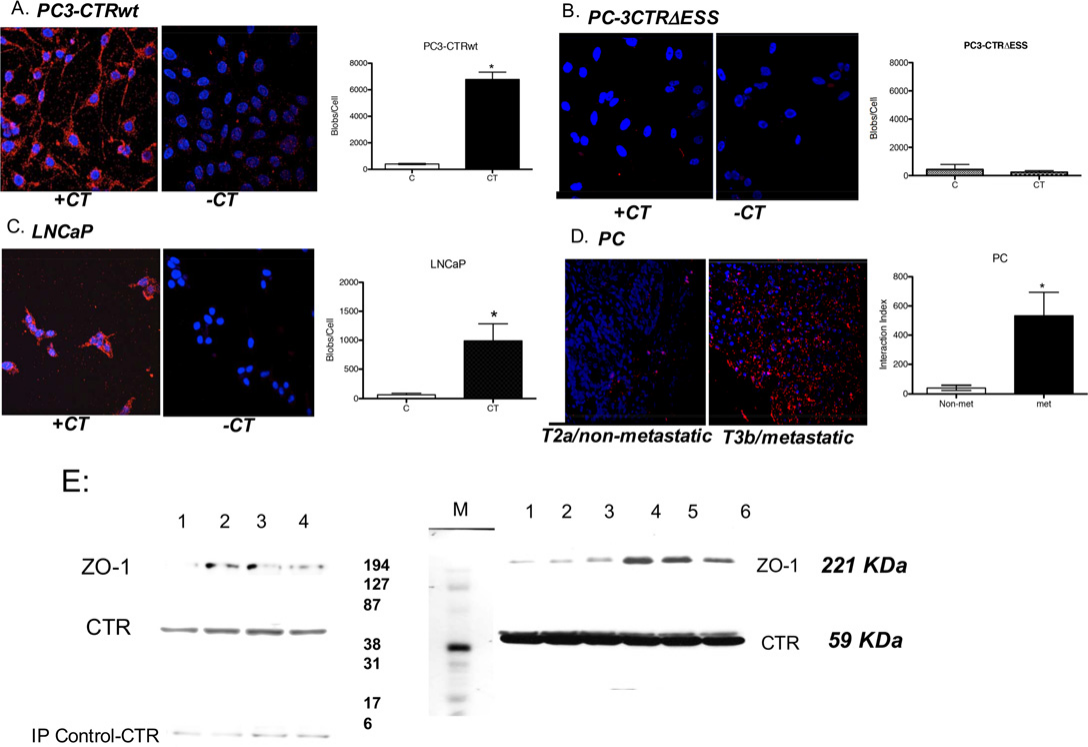
Detection of CTR-ZO-1 interaction in PC cell lines and tumor specimens. **A:** Representative micrographs of CTR-ZO-1 interactions in PC-3CTRwt cells in the absence/presence of CT stimulation for 30 seconds. Each red dot represents an interaction between activated CTR and ZO-1, detected by *in situ* PLA^™^ using the Duolink^®^ kit. Nuclei are stained with DAPI (blue). **The graph** presents quantitative data of CTR-ZO-1 interactions in three separate, identical experiments (mean ±SEM for n = 6, p<0.001, paired t-test). **B**: Representative micrographs of CTR-ZO-1 interactions in PC-3CTR**∆**ESS cells as described in A. **The graph** presents quantitative data of three separate, identical experiments described in Fig 7B (mean ±SEM for n = 6). **C**: Representative micrographs of CTR-ZO-1 interactions in LNCaP cells as described in A. **The graph** presents quantitative data of three separate, identical experiments described in Fig 7C (mean ± s.e.m. for n = 6, *p<0.001, paired t-test). **D**: Representative micrographs of CTR-ZO-1 interactions in PC specimens of metastatic (T3bN0M1; Gleason Score 10; Stage III, Prostate Adenocarcinoma, Malignant) and non-metastatic tumors (T2aN0M0; Gleason Score 6; Stage II, Prostate Adenocarcinoma). **The graph** presents quantitative data (mean ±SEM for n = 3 of each condition; *p<0.001, paired t-test). **E.** Snap-frozen human prostate tumor specimens were homogenized (10 mg by weight), and same amount of clarified supernatant was used for immunoprecipitation of CTR from all tumors. CTR IPs were fractionated on SDS-PAGE and ZO-1 was detected by immunoblotting (Rabbit Anti-ZO-1 serum, Invitrogen, Life Technologies, Grand Island, NY). The blots were normalized by the immunoprecipitation antibody (Rabbit Anti-CTR serum, Abcam, Cambridge, MA). The bands on the left side of molecular weight markers were from frozen prostate tissues (1- BPH; 2–4 Prostate Adenocarcinoma localized, Gleason Score 6–7). The bands on the right side of molecular weight markers were also from frozen prostate tissues- 1–3 were localized tumors (T2cN0M0; Gleason Scores 6–7) and 4–6 were metastatic (T3aN0Mx or greater; Gleason Scores-7-9). The tissues on the right side were limited in availability, and we could not do IP controls. The amount of tissues was limited for just one experiment. Therefore, we obtained multiple tissues to perform this experiment.

CTR-ZO-1 interactions in unmodified cells or tissues were examined by an *in situ* proximity ligation assay (PLA assay). Specificity of the assay was tested by replacing either goat anti-CTR or rabbit anti-ZO-1 or both antisera with isotypic control sera. No interaction signal (red dot) was detected (S1 Appendix, S2A–S2C Fig). Next, we examined CTR-ZO-1 interaction in PC-3CTRwt cells (Fig 7A). Each dot in the Figure reveals a single CTR-ZO-1 interaction. Some CTR-ZO-1 interactions were observed in the absence of exogenous CT stimulus, possibly due to activation of CTR by endogenous CT secretion [30]. However, stimulation with 50 nM CT led to eighty-fold increase in these interactions in 30 seconds, and every cell responded to CT stimulation (Fig 7A). Again, no CTR-ZO-1 interactions were observed with PC-3CTR∆ESS cells with or without CT (Fig 7B). Next, we tested this phenomenon in LNCaP cells, which are androgen-responsive and endogenously express CTR but not CT [30]. Unstimulated LNCaP cells did not display any CTR-ZO-1 interaction. When stimulated with CT (50 nM, 30 seconds), all cells responded with strong interaction between endogenous CTR and ZO-1 (Fig 7C). We then examined non-metastatic and metastatic PC specimens. CTR-ZO-1 interactions were observed in PC specimens, and the number of interactions in metastatic PC (tumor stage T3bN0M1) was thirteen-fold greater than non-metastatic PC (tumor stage T2aN0M0) (Fig 7D). To biochemically validate the results of Fig 7D, CTR was immunoprecipitated from lysates of snap-frozen, but otherwise unprocessed, human prostate tumors. As depicted in Fig 7E, ZO-1 was co-immunoprecipitated by CTR in all snap-frozen tumors. The results displayed relatively large variations in the amount of co-immunoprecipitated ZO-1 from different tumors. Considering the limitations with regard to the availability of tumor tissues (less than a few milligram by weight), it has not been possible to investigate if the variation in ZO-1 co-precipitation is due to the loss in CTR-ZO-1 interaction, processing of tissues for analysis, potential degradation while harvesting of the tissues or due to variations in cellular ZO-1 content in tumors. Nevertheless, the results provide the biochemical evidence for the occurrence of CTR-ZO-1 association in human prostate tumors, and validate the results of Fig 7D. Both, CTR and ZO-1 antisera for this experiment were checked for specificity (S1 Appendix, S2A–S2C Fig).

### CTR-C PDZ-binding motif is required for the metastasis of orthotopic xenografts

**Fig 8:**
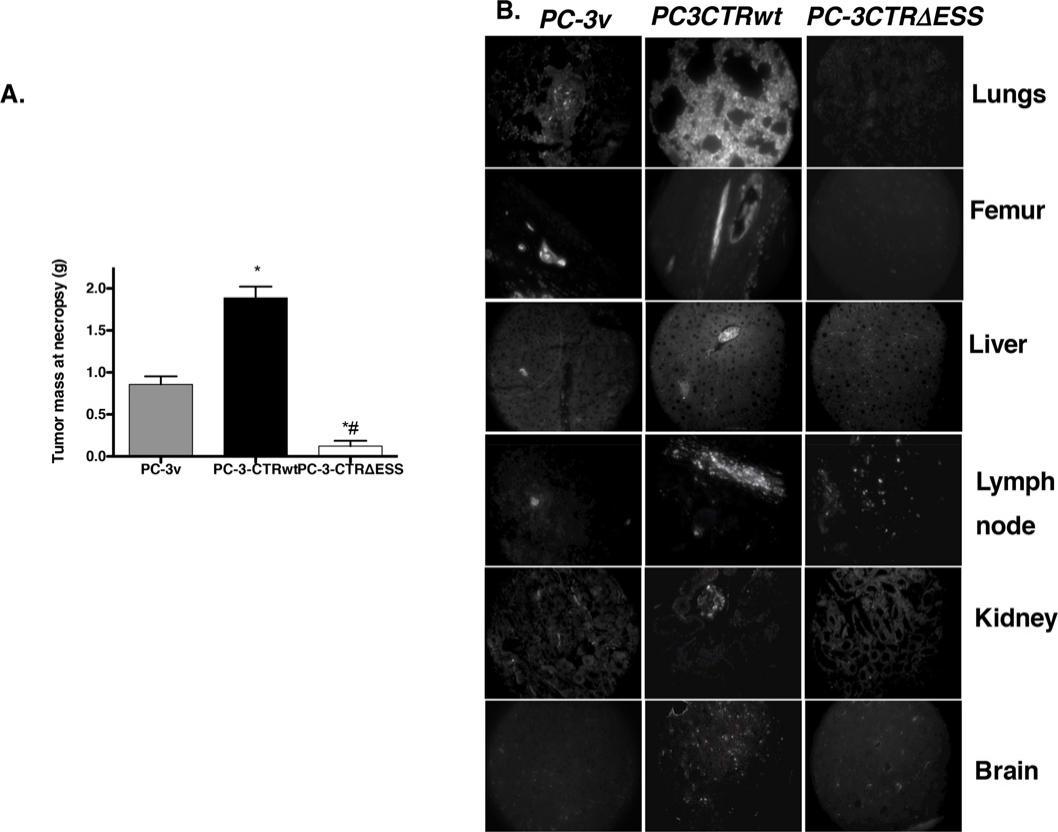
Orthotopic tumors and distant metastases formed by PC-3v, PC-3CTRwt and PC-3CTR∆ESS cells in nude mice. Red Fluorescent Protein (RFP)-expressing PC- 3 cells were transfected either with vector (v), CTR-wt or CTR-**∆**ESS constructs. The cells (1x10^6^) were orthotopically implanted into the prostate of nude mice (n = 6 per group). Animals were sacrificed 8 weeks after implantation, and prostate tumors were weighed (Fig # 8A); the indicated organs were removed from mice and observed directly under fluorescent microscope. The images were captured at 100X magnification (Fig 8B). *p<0.01 (PC-3v vs PC-3CTRwt); ^p<0.01 (PC3CTR∆ESS vs PC-3CTRwt); one-way ANOVA and Newman-keuls test.

**Fig 9:**
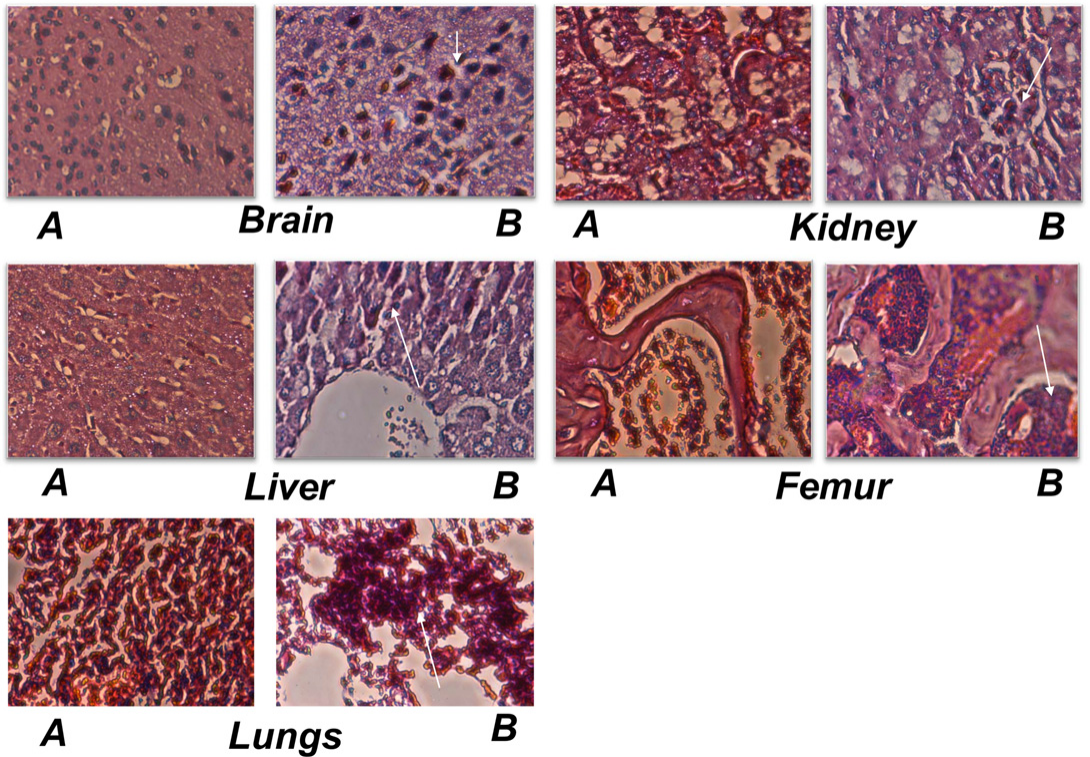
H&E sections of organs with metastases. Representative photomicrographs of H&E stained tissues of nude mice implanted with PC-3-CTR-wt (A) and PC-3-CTR∆ESS cells (B) as described in Fig 8. Magnification 400X. Micrometastases indicated by arrows.

We next examined the role of CTR-C PDZ-binding motif on tumor metastasis of PC-3 cells in the orthotopic nu/nu mouse model. As expected the PC-3 cell line, which lacks CTR, displayed only moderate orthotopic growth and metastasis in select organs (Fig 8A and 8B, Tables 2 and 3). PC-3-CTRwt cells formed significantly larger orthotopic tumors and micrometastases in multiple organs including lymph nodes, femur and lungs. In contrast, PC-3-CTR∆ESS-derived orthotopic tumors were comparable to those of PC-3V, and no micrometastases were observed in all tested organs except two out of six mice showed minor lymph node involvement (Tables 2 and 3). H&E sections of these tumors are presented in Fig 9.

**Table 2:** Micrometastases of PC-3 cells in host organs.

Organs	PC-3v	PC3-CTRwt	PC3-CTR∆ESS
Seminal Vesicles	++	+++	-
Testes	++	+++	-
Lymph nodes	++	++	+
Femur	+	+++	-
Lungs	+	++	-
Liver	+	++	-
Mesentary	+	++	-
Kidneys	+	++	-
Brain	-	-	-

**Table 3:** Frequency of Metastases in host organs.

Organs	PC-3v	PC3-CTRwt	PC3-CTR∆ESS	Number of Animals (n)
Seminal Vesicles	5	6	0	6
Testes	3	6	0	6
Lymph nodes	6	6	2	6
Femur	2	4	0	6
Lungs	1	3	0	6
Liver	2	2	0	6
Mesentary	1	1	0	6
Kidneys	1	2	0	6
Brain	0	0	0	6

It has been known that TJs are critical components of the epithelial barrier implicated in the maintenance of tissue integrity as well as the coordination of cell signaling and membrane trafficking [33]. The disruption or loss of TJs has been correlated with cell dissociation and acquisition of invasive phenotype in several human cancers [34–36]. Present results suggest that the activated CTR directly destabilizes TJs, and that the loss of TJs precedes its proinvasive and metastatic actions on prostate cancer cells. These results thus add temporal sequence to the present evidence that the loss of epithelial cell polarity is often associated with epithelial to mesenchymal transition [36].

Overlay assay identified that PDZ3 domain of ZO-1 is the site of its interaction with CTR-C tail. Multiple functional assays also confirmed that only the deletion of ZO-1-PDZ3 abolishes CTR-mediated TJ destabilization as well as invasion. It has been reported that ZO-1 binds to claudins via PDZ1 domain; to ZO-2 or ZO-3 via its PDZ2; to occludin through GK domain; and to actin via its–COOH terminus [37,38], leaving PDZ3 open for binding to CTR-C tail. Considering that the mutation of PDZ-binding ligand of CTR prevented metastasis of orthotopic xenografts, present results not only open up interesting possibilities to examine the role of CTR in modifying ZO-1 and/or its scaffold proteins, but also an experimental model to systematically examine the role of each of these components individually in prostate cancer metastasis.

Functionally, transmembrane proteins of TJs like claudins and occludin are shown to mediate cell-cell adhesion, whereas cytosolic TJ plaque proteins such as ZO-1 link transmembrane proteins to cytoskeleton [33,39]. ZO-1 is a multi-domain scaffolding protein that recruits signaling proteins such as protein kinases, phosphatases, small GTPases and transcription factors to the TJs [40]. Our results suggest that ZO-1 recruits CTR through its PDZ3 domain, and localizes the activated receptor within the TJ space. As a result, the effector molecules of CTR may juxtapose with ZO-1 scaffold, enabling CTR to activate its effectors in the close proximity of ZO-1 and its scaffold proteins. Based on our earlier studies, CTR-activated signaling events that are required for CTR-induced invasion include the activation of PKA [5,8,11,14]. Present results show that both, CTRwt and CTR∆ESS activate PKA with equal potency, but only CTRwt destabilizes TJs and increases invasion and metastasis. Thus, it appears that the capture of CTR by ZO-1 through the PDZ interaction brings CTR and its effectors such as PKA in the TJ domain. This may enable the CTR activated PKA to phosphorylate either ZO-1 and/or other proteins in its scaffold. This PKA-mediated phosphorylation of key proteins may be the trigger for collapse of TJs and subsequent events leading to EMT. Additional studies are in progress to examine this possibility.

CTR-ZO-1 interaction may have an important pathophysiological significance in prostate cancer progression. Earlier, we reported that CT and CTR transcripts are differentially localized in benign and malignant prostates. In benign prostates, CT and CTR were localized in basal epithelium. In contrast, CT and CTR mRNAs were detected in pseudoacini of prostate cancer, which lacks basal cells [30]. It has been shown that basal epithelial cells lack TJs, while luminal cells display their presence [41]. Based on this evidence, it appears that the lack of ZO-1 in normal basal epithelium may prevent CTR-ZO-1 interaction. In contrast, the appearance of TJs in pseudoacini of malignant prostates may facilitate CTR-ZO-1 interaction and subsequent events associated with invasion and metastasis. Present results that CTR-ZO-1 interaction is critical for proinvasive CTR actions explain the present inconsistency in prostate cancer pathophysiology that though CTR is present in normal basal and malignant epithelia, it becomes proinvasive only in malignant epithelium.

## Conclusions

In conclusion, we report several novel findings. First, we demonstrate the interaction between CTR-C binding motif and PDZ3 domain of ZO-1. Second, we demonstrate that CTR-ZO-1 interaction is critical for prometastatic actions of CTR on PC cells. Third, we demonstrate that both, CTRwt and CTR-∆ESS, activated effector molecules with almost equal potency. However, CTR-mediated proinvasive actions occurred only when CTR interacted with ZO-1, suggesting that the location of CTR-mediated signaling events is the critical factor for TJ destabilization and invasion. Fourth, we demonstrate the pathological significance of this event in prostate cancer metastasis. Finally, this experimental model opens up the possibility of identifying early signaling events associated with prostate cancer metastasis, may also provide a new target(s) for the treatment of advanced PCs, and a platform for the discovery of new anti-cancer agents.

## Supporting Information

S1 Appendix Authentication of Cell lines. (XLS)

S1 Fig Characterization of PC-31 cell line (A-D) and antisera.
(EPS)

S2 Fig (A-C).
(EPS)
